# Feeding Behavior and Ecological Significance of *Craspedacusta sowerbii* in a Freshwater Reservoir: Insights from Prey Composition and Trophic Interactions

**DOI:** 10.3390/biology14060665

**Published:** 2025-06-07

**Authors:** Hailong Yan, Yu Wang, Mengyao Wu, Yuying Li, Wanping Wang, Dongliang Zhang, Jingjing Guo, Nicola Fohrer, Bailian Larry Li

**Affiliations:** 1 International Joint Laboratory of Watershed Ecological Security for Water Source Region of Middle Route Project of South-North Water Diversion in Henan Province, College of Water Resource and Modern Agriculture, Nanyang Normal University, Nanyang 473061, China; nynuhailong@163.com (H.Y.); 2023086001022@nynu.edu.cn (M.W.); 82294011@nynu.edu.cn (W.W.); 15290736702@163.com (D.Z.); 18437928059@163.com (J.G.); nfohrer@hydrology.uni-kiel.de (N.F.); bai-lian.li@ucr.edu (B.L.L.); 2 Henan Academy of Fishery Sciences, Henan Academy of Agricultural Sciences, Zhengzhou 450044, China; 18624381963@163.com; 3 Department of Hydrology and Water Resources Management, Kiel University, 240980 Kiel, Germany; 4 Department of Botany and Plant Sciences, University of California, Riverside, CA 92521, USA

**Keywords:** *Craspedacusta sowerbii*, Danjiangkou Reservoir, feeding behavior, ecological role, aquatic food webs

## Abstract

This study investigates the feeding behavior and ecological role of *Craspedacusta sowerbii* in the Danjiangkou Reservoir, a crucial freshwater source in central China. Through in situ cultivation, microscopic examination, and amplicon sequencing analysis, we identified the primary food sources of *C. sowerbii* within the reservoir’s aquatic food web. Our results indicate that *C. sowerbii* predominantly consumes zooplankton, specifically rotifers, copepods, and cladocerans, while phytoplankton is ingested less frequently and often remains undigested. Amplicon sequencing data further confirms that the prey composition of *C. sowerbii* is enriched in zooplanktonic communities compared to phytoplanktonic communities. Our findings suggest that *C. sowerbii* plays a significant role in regulating plankton populations and shaping the planktonic community structure in the Danjiangkou Reservoir, thereby contributing to the ecosystem’s functions and trophic dynamics. This study enhances our understanding of the feeding ecology of *C. sowerbii* and highlights its potential as a bioindicator species for assessing freshwater ecosystem health and monitoring water quality.

## Simple Summary

This study delves into the eating habits and ecological importance of a freshwater jellyfish, *Craspedacusta sowerbii*, in China’s vital water source, the Danjiangkou Reservoir. The main question we sought to answer was what this jellyfish eats and how its diet might influence the reservoir’s ecosystem. Our goal was to pinpoint its primary food sources and its place within the aquatic food chain. We gathered samples of the jellyfish and the surrounding water it inhabits, employing microscopes and DNA sequencing to examine its diet. Our findings reveal that *C. sowerbii* primarily dines on microscopic animals known as zooplankton, such as rotifers and copepods, rather than on plant-like phytoplankton. This insight is vital because it sheds new light on how *C. sowerbii’s* diet can regulate zooplankton in the aquatic ecosystem, thereby impacting the broader food web and water quality. Understanding its role aids us in more effectively managing and safeguarding biodiversity in freshwater ecosystems, ensuring the well-being of this species and the overall health of these vital habitats.

## 1. Introduction

Aquatic organisms, including phytoplankton, zooplankton, fish, cnidarians, and various other aquatic life forms, play numerous crucial roles in freshwater ecosystems, with their contribution to nutrient cycling being particularly significant [1,2]. Essential nutrients such as carbon, nitrogen, and phosphorus are indispensable for maintaining water quality and supporting the growth of all aquatic organisms [3]. Through photosynthesis, phytoplankton absorbs carbon dioxide and assimilates dissolved mineral nutrients, particularly nitrogen and phosphorus, thereby facilitating the biological sequestration of these nutrients into organic forms [2,4]. This process is pivotal in mitigating eutrophication, preserving water clarity, and maintaining overall water quality [5]. The organic nutrients sequestered by phytoplankton are subsequently transferred to higher trophic levels through zooplankton grazing, thereby forming an intricate aquatic food web [6,7]. Phytoplankton and zooplankton constitute the foundation of the aquatic food chain, while cnidarians primarily prey on them, ultimately serving as a food source for predators at higher trophic levels [8]. The interactions among these aquatic organisms not only sustain the dynamics of the food chain and maintain ecological balance but also enhance nutrient cycling, contribute to stabilizing water quality, and promote ecological rehabilitation [9]. Consequently, the conservation and management of aquatic biological resources are imperative for preserving the biodiversity of these aquatic lives and the health and stability of the aquatic ecosystems [10,11].

The Danjiangkou Reservoir is a large freshwater reservoir located in central China [12], serving as a critical water source for both the region and the wider surrounding areas, including the water supply for Beijing, via the South-to-North Water Diversion Project [5,13,14]. The reservoir was created by the construction of the Danjiangkou Dam on the Han River, which has significantly altered the local hydrology and created a unique aquatic environment [15,16]. Research on the ecology of the Danjiangkou Reservoir has primarily focused on the biodiversity and water quality, examining how the introduction of the dam and subsequent changes to the water flow and habitat have influenced species composition and the functioning of the reservoir’s ecosystem [17,18,19]. Additionally, the Danjiangkou Reservoir supports a diverse array of aquatic life, including various species of phytoplankton, zooplankton, bacterioplankton, and the planktonic fungal community [13,15,20]. A total of 136 taxonomic units of planktonic algae were identified in the Danjiangkou Reservoir, with the dominant taxa belonging to the phyla Chlorophyta, Bacillariophyta, and Cyanophyta [14]. Furthermore, 65 zooplankton taxa were recorded, with Rotifera exhibiting the highest taxonomic richness among these groups [21]. More than 20,000 bacterioplankton sample bands were identified through high-throughput sequencing in the Danjiangkou Reservoir, with Proteobacteria, Actinobacteria, and Bacteroidetes accounting for 71.78% to 96.98% of the total population [22]. The composition of the planktonic fungal community in the Danjiangkou Reservoir was investigated using Illumina MiSeq sequencing, revealing that the community was primarily composed of Ascomycota, Rozellomycota, Basidiomycota, Chytridiomycota, and Zygomycota, encompassing 294 genera of planktonic fungi, thereby demonstrating the rich diversity of this community [15]. Given its importance as a water supply, monitoring biodiversity and water quality has been a critical aspect of research. However, the trophic dynamics of the aquatic food web in the Danjiangkou Reservoir are still poorly understood [23], despite their significant role in nutrient cycling and energy transfer, both of which are essential for evaluating the health and stability of the ecosystem.

*Craspedacusta sowerbii* (Lankester, 1880) is a freshwater hydrozoan classified within the phylum Cnidaria and the class Hydrozoa [8,24]. Originally native to the Yangtze River in China, it has since disseminated globally, except in Antarctica [25,26,27,28]. Despite its widespread occurrence in freshwater habitats, such as clean rivers, lakes, and ponds, *C. sowerbii* is highly sensitive to water quality, necessitating unpolluted conditions for its survival. This sensitivity renders its presence in freshwater ecosystems a reliable indicator of good water quality [29,30,31]. According to reports from local newspapers and residents, the Danjiangkou Reservoir provides a conducive habitat for *C. sowerbii*, indicating a stable aquatic environment within the reservoir. In addition to suitable environmental conditions, the establishment of *C. sowerbii* also depends on the availability of fundamental niche requirements, including adequate food resources [8,24,32]. Therefore, identifying the specific food sources that support the growth and reproduction of *C. sowerbii* in the Danjiangkou Reservoir is crucial for ensuring the long-term ecological stability of the species. However, the specific prey of *C. sowerbii* remains largely unidentified [33,34]. It was reported that the stomach contents of *C. sowerbii* included most of the taxa abundant in the zooplankton, which appeared to be a positive selectivity for the active copepod *Ceriodaphnia* [35]. The presence of *C. sowerbii* significantly altered zooplankton communities and aquatic food webs via both direct predation and incidental mortality [36]. Lüskow pointed out that cladocerans and copepods serve as suitable prey for *C. sowerbii* [37], implying that this species holds greater significance within freshwater food webs than previously acknowledged. 

In the present study, amplicon sequencing technology was employed to analyze the prey communities of *C. sowerbii* within the Danjiangkou Reservoir. To achieve this, environmental samples from the reservoir’s habitat and intestinal biota samples of *C. sowerbii* were collected separately for amplicon sequencing analysis. By integrating the microscopic observations of *C. sowerbii’s* stomach contents with the sequencing results, this study provides a detailed characterization of the dietary components of this species in the Danjiangkou Reservoir. The objectives of this study were to analyze the food sources and feeding characteristics of *C. sowerbii*, to elucidate its trophic position within the aquatic food web, and to infer the potential roles that *C. sowerbii* plays in maintaining aquatic ecological functions within the reservoir.

## 2. Materials and Methods

### 2.1. Cultivation of C. sowerbii and Sampling

**Figure 1:**
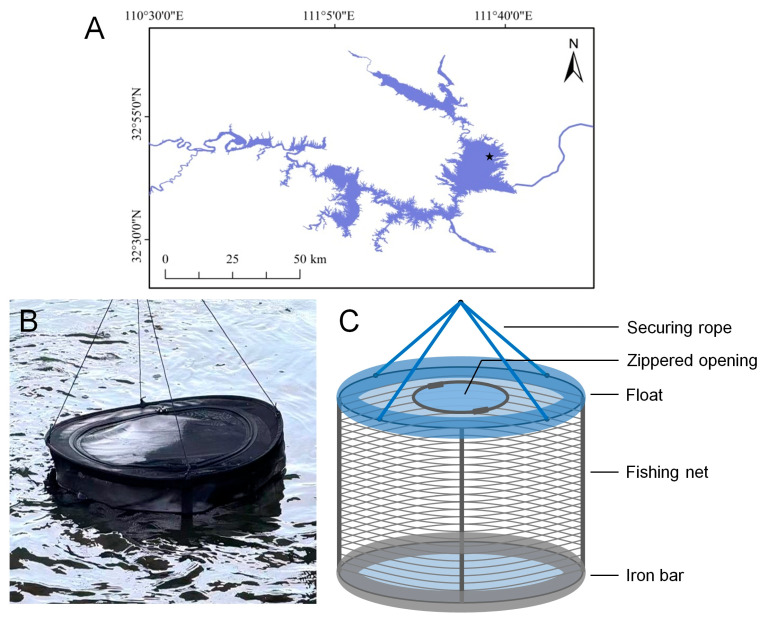
The sampling point for in situ cultivation of *C. sowerbii.* (**A**) Overview of the Danjiangkou Reservoir and the sampling point (marked with a star). (**B**) The fishing net used to cultivate *C. sowerbii.* (**C**) Structure of the fishing net.

As reported, the first seasonal occurrence of *C. sowerbii* was in July, with the latest occurrence in October, peaking in August and September [38]. This experiment was conducted in the middle of September. The sampling point was located in the northeast part of the Danku, about 10 m away from the lake shore (32°46′ N, 111°38′ E, Figure 1A, marked with a star). More than 300 individuals of *C. sowerbii* were collected from a natural pond using a 500 mL plastic ladle and transferred into a 10 L plastic bucket. The collected specimens were then transported to the sampling point, where they were cultured in a cylindrical fishing net measuring 40 cm in diameter and 60 cm in height (Figure 1B). The net featured a mesh size of 2 mm, allowing plankton and microorganisms to freely enter and exit (Figure 1B). The lower end of the net was attached to iron bars, while the upper end was equipped with floats, allowing the net to remain vertically suspended in the water (Figure 1C). Simultaneously, the environmental biological samples were collected from the sampling point with a total volume of 10 L. These biological samples were then filtered through a planktonic net featuring a mesh diameter of approximately 200 μm to eliminate impurities. After filtration, the samples were concentrated to 10 mL using a centrifuge. The environmental samples were collected prior to incubating *C. sowerbii* to characterize the background biotic community structure at the experimental sampling site in the Danjiangkou Reservoir. These samples also served as a control group for comparison with the food composition of *C. sowerbii*. As for the *C. sowerbii* samples, it is challenging to culture them, and as the adult life cycle is notably short, the vitality of most adult *C. sowerbii* specimens declined significantly after one week or more of culture. Consequently, they were collected at 1, 3, and 5 days post-incubation (DPI) to ensure that the observed feeding behaviors were representative. Meanwhile, the environmental samples and the *C. sowerbii* samples collected at different time points were utilized for microscopic observation and amplicon sequencing, respectively.

### 2.2. Microscopic Observations and Photographing

Through prolonged experimental observations, we found that the feeding behavior of *C. sowerbii* was most active at 3 DPI. Consequently, we selected this timeframe to observe and record their specific feeding processes under a microscope. Over 100 active individuals of *C. sowerbii* were collected for microscopic observations at 3 DPI. Each individual was carefully picked up using a pipette with a diameter of approximately 10 mm, and then the *C. sowerbii* samples were placed on a slide. With its gastric cavity exposed manually, each specimen was observed under a microscope (Olympus BX53, equipped with a Canon 80D camera, Tokyo, Japan). Photographs or videos were recorded immediately when organisms, such as phytoplankton or zooplankton, were detected within the gastric cavity of *C. sowerbii*. For observations of living *C. sowerbii*, photographs were taken with a Canon 80D camera, equipped with a 50 mm macro lens (Canon Ltd., Tokyo, Japan). Photographs or videos were edited to enhance contrast using Adobe Premiere Pro or Adobe Photoshop (Adobe Systems Incorporated, San Jose, CA, USA).

### 2.3. Amplicon Sequencing and Analyzing

More than 50 active individuals of *C. sowerbii* inside the fishing net were retrieved at 1, 3, and 5 DPI, respectively. Each individual *C. sowerbii* was washed with sterile water, the gastric cavity was dissected, and it was placed in liquid nitrogen immediately for the next step of conducting amplicon sequencing analysis. Amplicon sequencing was carried out by Novogene Co., Ltd., a domestic company, Tianjin, China, following procedures that encompassed sample quality control, amplicon generation, PCR product quantification and qualification, library preparation, and sequencing according to the company’s standard protocol. To delineate the composition of prokaryotes and eukaryotes, respectively, specific primers with barcodes were employed to amplify the 16S rRNA gene (using primers 515F-GTGCCAGCMGCCGCGGTAA and 806R-GGACTACHVGGGTWTCTAAT) and the 18S rRNA gene (using primers 528F-GCGGTAATTCCAGCTCCAA and 706R-AATCCRAGAATTTCACCTCT), respectively. Paired-end reads were assigned to samples based on their unique barcode and truncated by cutting off the barcode and primer sequence, and then they were merged using FLASH (Version 1.2.11). Quality filtering on the raw tags was performed using fastp (Version 0.23.1) to obtain high-quality clean tags. The tags were compared with the reference database to detect chimera sequences, and effective tags were obtained by removing the chimera sequences with the vsearch package (Version 2.16.0). For the effective tags obtained previously, the denoising process was performed with DADA2 or deblur module in QIIME2 software (version 2023.2) to obtain initial Amplicon Sequence Variants (ASVs). Species annotation was performed using QIIME2 software, and the annotation database was the Silva Database (Silva 138.1).

The relative abundances of food organisms in the gastric cavity of *C. sowerbii* and the surrounding environmental samples were determined through amplicon sequencing analysis. The representative sequences of ASVs were aligned against the annotation database to identify their closest taxonomic assignments with a confidence threshold of ≥80%. Each ASV was annotated to confirm its corresponding species name. The total number of sequences associated with each ASV was counted to determine the abundance of each species and its corresponding sequence count. The relative abundance of each species was calculated by dividing the sequence count by the total number of sequences. Cumulative bar charts were then generated. In these charts, each bar represents a different sample, and each color corresponds to a species. The percentage of a specific color within each bar indicates the proportion of that species relative to the total identified species. These bar charts enable an intuitive comparison of species composition across different samples.

### 2.4. Measurement of the Water Physicochemical Variables

Two liters of the surface water from the sampling point were collected, and the following water physicochemical variables, including permanganate index (COD_Mn_), ammonium nitrogen (NH_4_^+^-N), nitrate nitrogen (NO_3_^−^-N), total nitrogen (TN), total phosphorus (TP), and Chlorophyll a (Chl *a*), were analyzed according to the method described by Li [16]. Additionally, water temperature (WT), pH, dissolved oxygen (DO), electrical conductivity (EC), and oxidation-reduction potential (ORP) were measured in situ using a portable YSI 6920 instrument (YSI Inc., Yellow Springs, OH, USA). Water transparency (SD) was determined using the Secchi disk method, as described by Wu [14]. Meteorological parameters, including air pressure, humidity, and wind conditions, were measured using a hand-held anemometer–barometer.

## 3. Results

### 3.1. Environmental Variables of the Sampling Point in the Danjiangkou Reservoir

The physicochemical variables of the water in the sampling point of the Danjiangkou Reservoir were measured during the sampling period. The mean value of environmental variables, including WT, pH, DO concentration, ORP and EC, TN and TP, concentrations of COD_Mn_ and Chl *a*, SD, and meteorological variables, including air pressure, humidity, and wind speed, are listed in Table 1. The physicochemical variables of the analyzed water bodies comprehensively comply with the Class II standards for surface waters, as stipulated in China’s Environmental Quality Standard for Surface Water (GB 3838-2002) [14], demonstrating that the Danjiangkou Reservoir sustains excellent water quality and provides a favorable habitat for *C. sowerbii*.

### 3.2. The Feeding Behavior and Anatomy of C. sowerbii

**Figure 2:**
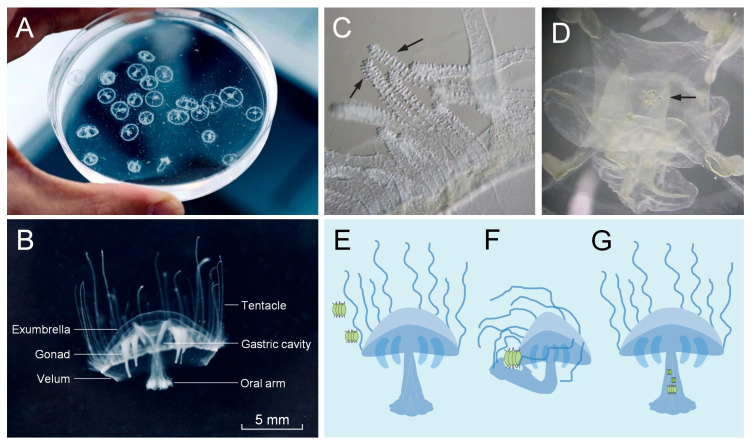
Behavioral observations of live *C. sowerbii* specimens. (**A**,**B**) In situ behavioral observations of *C. sowerbii*. (**C**) Tentacles of *C. sowerbii* (arrows). (**D**) The suspected prey in the gastric cavity of *C. sowerbii* (arrow). (**E**) Phytoplankton approached the tentacles of *C. sowerbii*. (**F**) *C. sowerbii* grasped the phytoplankton by curling its tentacles and simultaneously bending its oral arm to envelop the prey. (**G**) Phytoplankton stored in the gastric cavity of *C. sowerbii*.

Active specimens of *C. sowerbii* were collected from the sampling point at 3 DPI and were transferred to a 9 cm diameter Petri dish for microscopic observation (Figure 2A). Living individuals of *C. sowerbii* were observed using a camera equipped with a 50 mm macro lens (Figure 2B). The methods for observation and description of *C. sowerbii* were based on previous studies [27,39]. *C. sowerbii* exhibited an umbrella-shaped body with a diameter ranging from 1 to 2 cm. The body primarily consisted of six components, including the exumbrella, gonad, velum, tentacle, gastric cavity, and oral arm (Figure 2B). Among these, the exumbrella and velum were the principal structures constituting the body of *C. sowerbii* (Figure 2B). The gonad functioned as the reproductive organ, playing a crucial role in its reproductive processes. The oral arm served as both the feeding and excretory opening. The body edge was surrounded by numerous short tentacles covered with countless protuberances (Figure 2C, arrows), which are believed to assist in capturing prey [40]. Subsequently, the gastric cavity stored and digested the captured prey (Figure 2D, with an arrow pointing to a suspected prey).

Phytoplankton and zooplankton collected from the designated sampling point were utilized as prey for *C. sowerbii*, facilitating the observation of its feeding behavior. The results indicated that when a phytoplankton, for example, made contact with the tentacles of *C. sowerbii* (Figure 2E), the organism promptly grasped the phytoplankton by curling its tentacles and simultaneously bending its oral arm to envelop the prey (Figure 2F). Following this interaction, *C. sowerbii* sank to the bottom of the Petri dish and remained stationary for an extended period, absorbing the prey into its oral arm. Subsequently, *C. sowerbii* ascended to the water’s surface, with the ingested prey stored in its gastric cavity (Figure 2G).

### 3.3. Microscopic Observations of the Environmental Species Community and the Food Composition of C. sowerbii

**Figure 3:**
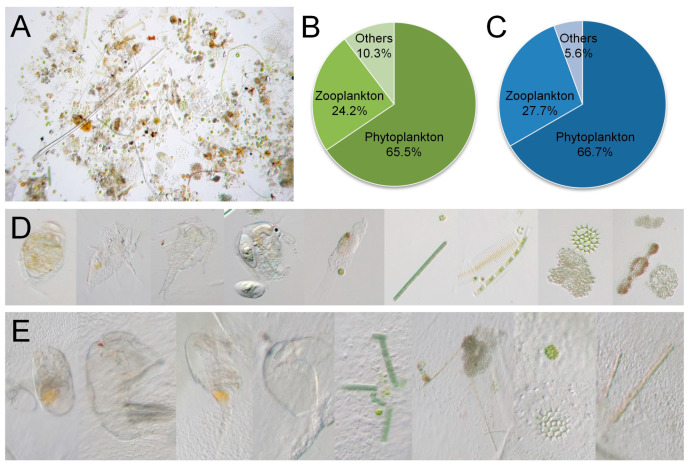
The environmental species community and food composition of *C. sowerbii*. (**A**) Concentrated water sample of the Danjiangkou Reservoir. (**B**) Percentage of the environmental species community. (**C**) Percentage of the species community in the gastric cavity of *C. sowerbii*. (**D**) Typical environmental species observed under a microscope. (**E**) Typical species community observed within the gastric cavity of *C. sowerbii*.

A total of ten liters of water collected from the sampling point were concentrated and subsequently observed under a microscope (Figure 3A). We identified nineteen species of phytoplankton, which accounted for 65.5% of the total, and seven species of zooplankton, comprising 24.2% of the total count, along with three additional species, including nematodes and larval stages of aquatic animals (Figure 3B). Microscopic dissection of the gastric cavity of *C. sowerbii* revealed a total of twelve species of phytoplankton, which represented 66.7% of the total prey count, alongside five species of zooplankton, accounting for 27.7% of the total, and one suspected prey item resembling fish eggs (Figure 3C). The typical environmental species communities observed in this study are illustrated in Figure 3D. These communities included various zooplankton species, such as rotifers, ciliates, copepods, and cladocerans, as well as phytoplankton species, including *Oscillatoria*, *Pediastrum*, *Microcystis*, and *Fragilaria* (Figure 3D), indicating the rich planktonic biodiversity within the Danjiangkou Reservoir. The species documented within the gastric cavity of *C. sowerbii* at 3 DPI were also listed. Among these, zooplankton such as rotifers, copepods, and cladocerans were identifiable through their digested remains (Figure 3E). In contrast, phytoplankton species, like *Oscillatoria*, *Pediastrum*, and *Microcystis*, although detected in the gastric cavity, remained undigested (Figure 3E). It cannot be excluded that some planktonic species were completely digested within the gastric cavity of *C. sowerbii*, rendering any remnants unrecognizable. Nevertheless, based on microscopic observations of the food composition within the gastric cavity of *C. sowerbii*, we concluded that the primary food source for *C. sowerbii* in the Danjiangkou Reservoir was zooplankton rather than phytoplankton.

### 3.4. Amplicon Sequencing Analysis of the Food Composition of C. sowerbii

Microscopic examinations of the food composition of *C. sowerbii* have inherent limitations; for example, they may fail to capture the full diversity of microbial communities consumed. However, amplicon sequencing provides a powerful tool for investigating the feeding ecology of *C. sowerbii*, particularly in detecting environmental microbial communities, including those that are low in abundance or unobservable using microscopic methods [41,42,43]. This molecular approach reveals a more comprehensive picture of the feeding patterns and trophic interactions within aquatic microbial networks [44,45]. Therefore, environmental samples procured from the experimental site, along with *C. sowerbii* specimens cultured for 1, 3, and 5 DPI at the same site, were subjected to amplicon sequencing analysis.

**Figure 4:**
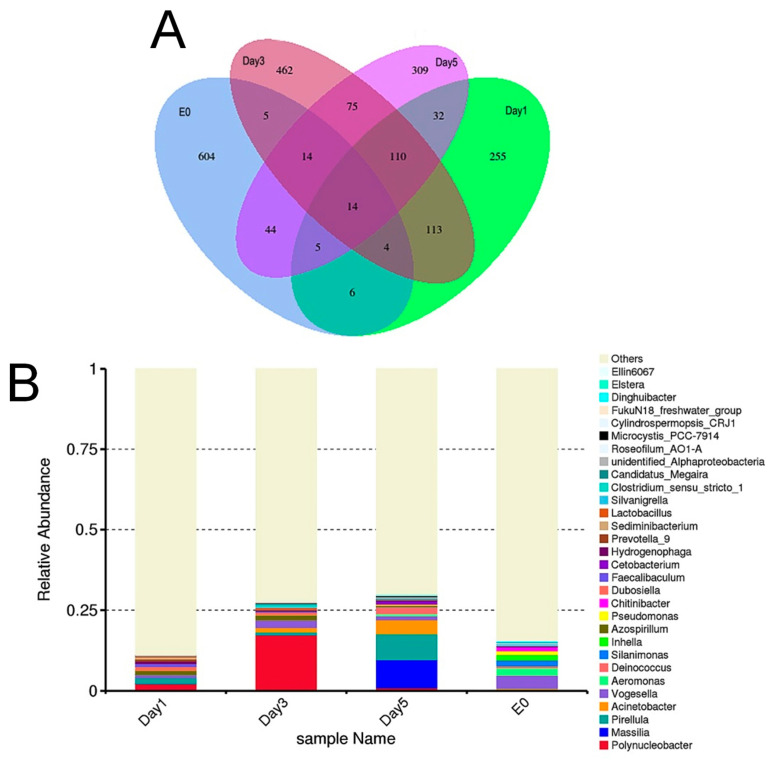
16S amplicon sequencing analysis comparing environmental samples with *C. sowerbii* samples. (**A**) Venn diagram analysis of the ASVs via 16S amplicon sequencing. (**B**) Bar plot showing the relative abundance of the top 30 genera identified via 16S amplicon sequencing.

**Figure 5:**
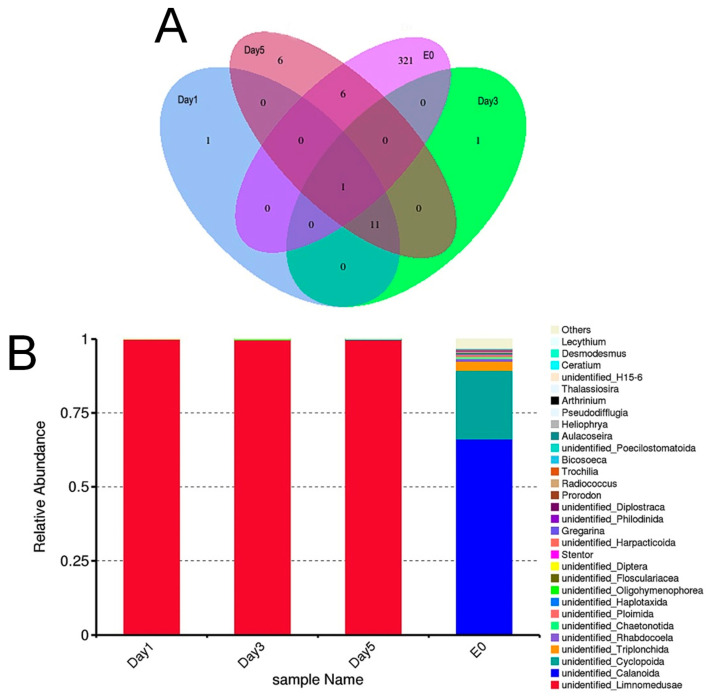
18S amplicon sequencing analysis comparing environmental samples with *C. sowerbii* samples. (**A**) Venn diagram analysis of the ASVs via 18S amplicon sequencing. (**B**) Bar plot showing the relative abundance of the top 30 genera identified via 18S amplicon sequencing.

A total of 696 and 323 ASVs were identified from the environmental samples (E0) through 16S (Figure 4A, and File S1) and 18S (Figure 5A, and File S1) amplicon sequencing, respectively. For the *C. sowerbii* specimens collected at 1, 3, and 5 DPI, 539, 797, and 603 ASVs were identified via 16S amplicon sequencing, respectively (Figure 4A, and File S1), while 13, 13, and 24 ASVs were identified via 18S amplicon sequencing, respectively (Figure 5A, and File S1). Among these, fourteen ASVs were present in all samples analyzed by 16S amplicon sequencing (Figure 4A, and File S1), whereas one ASV was detected in all samples analyzed by 18S amplicon sequencing (Figure 5A, and File S1).

The relative abundance of the top 30 genera, as determined through 16S and 18S amplicon sequencing, was also analyzed. For the environmental sample (E0), the five most abundant genera identified via 16S amplicon sequencing were *Vogesella*, *Aeromonas*, *Silanimonas*, *Inhella*, and *Pseudomonas* (Figure 4B). In contrast, the genera identified through 18S amplicon sequencing included unidentified_*Calanoida*, unidentified_*Cyclopoida*, unidentified_*Triplonchida*, unidentified_*Rhabdocoela*, and unidentified_*Chaetonotida*, respectively (Figure 5B). For *C. sowerbii* samples collected at 1, 3, and 5 DPI, the top 30 genera identified via 16S amplicon sequencing included *Polynucleobacter*, *Aeromonas*, *Pseudomonas*, *Inhella*, *Massilia*, *Acinetobacter*, *Candidatus Megaira*, *Alphaproteobacteria,* and *Azospirillum*. Notably, *Polynucleobacter* was the most abundant genus at 1 and 3 DPI, while *Massilia* was the most abundant at 5 DPI (Figure 4B). It is important to note that host DNA constituted the majority of the sequencing results, predominantly reflecting the sequence information from *C. sowerbii* itself as determined through 18S amplicon sequencing. In addition to *C. sowerbii*, the most abundant genera in the *C. sowerbii* samples were unidentified_*Oligohymenophorea* at 1 and 3 DPI and unidentified_*Cyclopoida* at 5 DPI, respectively (Figure 5B).

### 3.5. Beta Diversity Analysis of the Food Composition of C. sowerbii

**Figure 6:**
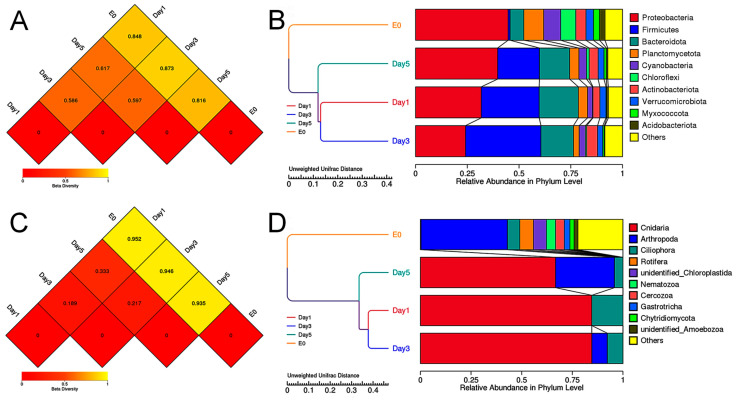
Beta diversity analysis comparing environmental samples and *C. sowerbii* samples. (**A**) Heatmap visualization of the Unweighted UniFrac Distance Matrix via 16S amplicon sequencing. (**B**) UPGMA clustering tree derived from 16S amplicon sequencing. (**C**) Heatmap visualization of the Unweighted UniFrac Distance Matrix via 18S amplicon sequencing. (**D**) UPGMA clustering tree derived from 18S amplicon sequencing.

In the beta diversity analysis, the Unweighted UniFrac Distance Matrix was utilized to assess the dissimilarity coefficient between two samples, with smaller values indicating reduced differences in species diversity. Under both 16S and 18S amplicon sequencing, the differences in species diversity between environmental samples (E0) and *C. sowerbii* samples collected at 1, 3, and 5 DPI were greater than those observed among the *C. sowerbii* samples themselves (Figure 6A,C). Notably, the smallest difference in species diversity was found between the *C. sowerbii* samples collected at 1 DPI and 3 DPI, followed by the samples collected at 3 DPI and 5 DPI (Figure 6A,C). These findings suggest that the feeding characteristics of *C. sowerbii* remain consistent across different sampling time points, while significant differences exist between the composition of its prey species and that of the surrounding environmental species.

To investigate the similarity among different samples, an Unweighted Pair-group Method with Arithmetic Mean (UPGMA) clustering analysis was conducted using the Unweighted UniFrac Distance Matrix. The clustering results were integrated with the relative abundance of species at the phylum level for each sample and presented visually. The results indicated that samples of *C. sowerbii* collected at 1, 3, and 5 DPI were more closely related to each other than to environmental samples (E0), as evidenced by both 16S and 18S amplicon sequencing analyses (Figure 6B,D). Furthermore, it is noteworthy that certain phyla detected in environmental samples, including Proteobacteria, Firmicutes, Bacteroidota, Planctomycetota, Cyanobacteria, Chloroflexi, Actinobacteriota, Verrucomicrobiota, Myxococcota and Acidobacteriota (identified via 16S amplicon sequencing), and Arthropoda and Ciliophora (identified via 18S amplicon sequencing), were consistently present in *C. sowerbii* samples collected at different time points (Figure 6B,D). This suggests that these phyla may constitute a fundamental food source for *C. sowerbii* in the Danjiangkou Reservoir.

## 4. Discussion

The feeding mechanism of *C. sowerbii* involves the utilization of its tentacles to capture prey and subsequently digest it. Observations indicate that when prey items, such as phytoplankton or zooplankton, came into contact with the tentacles, *C. sowerbii* curled its tentacles around the prey while simultaneously bending its oral arm to envelop and secure it (Figure 2). This behavior aligns with previous studies that have described the feeding strategy of *C. sowerbii* [39,40]. Once captured, the prey was stored and digested within the gastric cavity of *C. sowerbii*. Microscopic observations revealed that *C. sowerbii* can consume a variety of zooplankton and phytoplankton species (Figure 2 and Figure 3), demonstrating its adaptability and flexibility in prey selection. However, it primarily feeds on zooplankton rather than phytoplankton in the Danjiangkou Reservoir, which was evidenced by both microscopic observations of the contents within the gastric cavity (Figure 3) and amplicon sequencing analysis (Figure 4, Figure 5 and Figure 6). Microscopic examinations show that zooplankton species, including rotifers, copepods, and cladocerans, constitute a substantial portion of the identified prey items within the gastric cavity of *C. sowerbii* (Figure 3E). In contrast, while phytoplankton species were also detected, they were frequently found in an undigested state (Figure 3E), suggesting that *C. sowerbii* may exhibit a preference for or possess higher digestive efficiency towards zooplankton. The amplicon sequencing analysis provided further corroborates this finding, demonstrating that the gastric cavity of *C. sowerbii* exhibited a notably higher relative abundance of zooplanktonic communities compared to phytoplanktonic communities. These findings suggested that *C. sowerbii* actively preyed upon and primarily consumed zooplankton as its main food source within the Danjiangkou Reservoir. Previous studies on feeding habits have reported that *C. sowerbii* primarily feeds on zooplankton [35,36,37], which aligns with our findings. Beyond these findings, Deserti also reported that on the trophic ecology and diet of *Hydra vulgaris*, although algae dominated the diet in terms of abundance and frequency of occurrence, their volumetric contribution and nutritional value were almost negligible. They suggested that algae might enter the coelenteron incidentally or attach to the skeletons of invertebrate prey rather than being actively consumed for nutrition [46], which further supports our results.

Plankton are known to host a diverse array of microbial communities, including bacteria, fungi, and viruses [47,48]. When predators consumed plankton, they also ingested these microbial communities, which can be detected in the intestinal biota. Amplicon sequencing analysis successfully identified these plankton-associated microbial (PAM) communities in the gastric cavity of *C. sowerbii*, utilizing specific primers designed to amplify the 16S rRNA gene of prokaryotes and the 18S rRNA gene of eukaryotes. This approach allows for the detection of both bacterial and eukaryotic microbial communities [16]. In the present study, bacterial communities at the genus level were analyzed based on 16S amplicon sequencing (Figure 4). Among the top 30 genera shared between bacterial communities in environmental samples and those in the gastric cavity of *C. sowerbii*, a significant number of PAM communities were identified, which not only reflects their survival strategy but also embodied their ecological role within the Danjiangkou Reservoir (Figure 4). Among them, *Polynucleobacter* was ubiquitously detected in freshwater habitats, and its composition was highly associated with phytoplankton [49]. *Aeromonas* and *Pseudomonas* have been identified as algicidal bacteria exhibiting algicidal activity against cyanobacteria and green algae [50]. *Inhella* and *Massilia* have been suggested as new algicidal bacteria [51]. *Acinetobacter* has been recognized as an algae-associated beneficial bacterium that exhibited synergistic cooperation with algae to enhance nutrient removal efficiency [52]. *Candidatus Megaira* represents diverse symbionts of algae and ciliates, with the potential for defensive symbiosis [53]. Furthermore, *Alphaproteobacteria* have demonstrated the ability to promote the growth of green algae [54], while *Azospirillum* has been acknowledged as a bacterium that promoted algal growth [55]. These microbial communities were estimated to play important roles in shaping the food webs in the Danjiangkou Reservoir; they not only interacted with phytoplankton or zooplankton to serve as decomposers or nutrient transmitters but also completed the energy flow from primary producers to *C. sowerbii*.

The feeding behavior of *C. sowerbii* in the Danjiangkou Reservoir has been observed and analyzed, yielding valuable insights into its ecological role and trophic dynamics within the aquatic food web in the Danjiangkou Reservoir. Though *C. sowerbii* have traditionally been regarded as trophic dead-ends due to their limited number of predators within freshwater food webs, marine jellyfish are frequently preyed upon by fish, birds, turtles, and various invertebrates [8,56]. Given these insights, we hypothesize that *C. sowerbii* may support similarly predator populations in freshwater ecosystems. A conceptual model illustrates this role as follows. Phytoplankton in the reservoir generates primary productivity through photosynthesis, forming the base of the food chain. After that, a portion of the organic nutrients synthesized by phytoplankton is assimilated by PAM, while another portion is transferred to herbivorous zooplankton that graze on phytoplankton. The diet of zooplankton also includes a portion of PAM through suspension feeding. Subsequently, by preying on phytoplankton, PAM, and zooplankton, *C. sowerbii* ultimately recycles nutrients into its own body. As a predator in the aquatic food web, *C. sowerbii* plays a crucial role in controlling plankton populations and shaping the structure of the planktonic community [36,37,57]. By feeding on zooplankton, *C. sowerbii* helps to regulate the abundance and diversity of these organisms, which in turn influences the dynamics of the entire aquatic food web [35,36]. Furthermore, the presence of *C. sowerbii* in the Danjiangkou Reservoir contributes to an enhancement of the biodiversity of the local aquatic ecosystem. This increase in biodiversity serves as a significant factor, prompting both newspapers and local residents to regard it as a key indicator of favorable water quality.

While this study provides valuable insights into the feeding behavior and ecological role of *C. sowerbii* in the Danjiangkou Reservoir, it is essential to acknowledge the limitations of the current research. Firstly, the study was conducted at a single sampling point within the reservoir and at a specific time point in mid-September. This limited spatial and temporal scope may not fully capture the variability in *C. sowerbii’s* feeding behavior and prey selection throughout the entire reservoir and across different seasons. Future studies should aim to expand the spatial coverage by sampling from multiple locations within the Danjiangkou Reservoir and conducting long-term monitoring across different seasons. This will facilitate a more comprehensive understanding of how environmental factors, such as temperature, light availability, and nutrient concentrations, influence *C. sowerbii’s* feeding habits and ecological function within the reservoir. Additionally, comparative studies in other freshwater ecosystems with varying environmental conditions could further validate the generality of our findings and elucidate the broader ecological implications of *C. sowerbii’s* presence in freshwater habitats. Such research will not only enrich our knowledge of this fascinating species but also contribute to the effective management and conservation of freshwater ecosystems globally.

## 5. Conclusions

This study provides a comprehensive examination of the feeding behavior and ecological role of *C. sowerbii* in the Danjiangkou Reservoir, a vital water source located in central China. By integrating in situ cultivation experiments, microscopic examinations, and amplicon sequencing analysis, we elucidate that zooplankton, particularly rotifers, copepods, and cladocerans, constitute the primary dietary components for *C. sowerbii* within the reservoir’s aquatic food web. The amplicon sequencing data robustly corroborates these findings, demonstrating a significantly higher relative abundance of zooplanktonic communities compared to phytoplanktonic communities in the prey of *C. sowerbii*. Our findings underscore the pivotal role played by *C. sowerbii* in modulating plankton populations and shaping the planktonic community structure in the Danjiangkou Reservoir. By predominantly consuming zooplankton, *C. sowerbii* plays a crucial role in maintaining ecological equilibrium and trophic dynamics within the reservoir. Furthermore, the presence of *C. sowerbii* in the Danjiangkou Reservoir serves as an indicator of good water quality, highlighting its potential as a bioindicator species for assessing freshwater ecosystem health and monitoring water quality. Any significant change in the population size of *C. sowerbii* in a specific area suggests that the aquatic ecosystem in that region may have been impacted. For example, during the early stages of planning and preparation for the Three Gorges Dam on the Yangtze River, experts from the Institute of Hydrobiology, Chinese Academy of Sciences, realized that the dam’s construction might affect the habitat of *C. sowerbii*. As a result, they formulated a plan to rescue the *C. sowerbii* population in Zigui, its natural habitat. They implemented measures to relocate specimens to Beijing for heterogeneous breeding, aiming to preserve the species. However, these efforts have yielded limited results due to the absence of targeted breeding and rearing methods for *C. sowerbii*. In our study, we uncovered the feeding characteristics of *C. sowerbii*, which can provide new insights for better conserving this freshwater resource.

In conclusion, this study enhances our understanding of *C. sowerbii’s* feeding ecology and its significance within freshwater food webs. The findings provide valuable insights into the trophic interactions and ecological roles of *C. sowerbii* in the Danjiangkou Reservoir and suggest that future research on this species across diverse freshwater ecosystems may reveal broader ecological implications, thereby contributing to the effective management and conservation of freshwater resources worldwide.

## Figures and Tables

**Table 1:** Environmental variables of the sampling point in the Danjiangkou Reservoir.

Variables	Mean ± SD
WT (°C)	27.85 ± 1.28
pH	8.15 ± 0.37
DO (mg/L)	9.49 ± 0.61
ORP (mV)	160.43 ± 46.55
EC (μS/cm)	602.72 ± 50.01
TN (mg/L)	1.963 ± 0.52
TP (mg/L)	0.025 ± 0.01
COD_Mn_ (mg/L)	2.39 ± 0.52
Chl a (μg/L)	3.55 ± 1.38
SD (m)	2.21 ± 0.73
Air Pressure (kPa)	99.37 ± 3.60
Humidity (%)	87.44 ± 8.34
Wind (m/s)	1.14 ± 0.42

## Data Availability

The data are contained within the article.

## Supplementary Materials

The following supporting information can be downloaded at https://www.mdpi.com/article/10.3390/biology14060665/s1, File S1. Raw Data of the amplicon sequencing.
